# Respiratory motion correction of PET using MR-constrained PET-PET registration

**DOI:** 10.1186/s12938-015-0078-5

**Published:** 2015-09-18

**Authors:** Daniel R. Balfour, Paul K. Marsden, Irene Polycarpou, Christoph Kolbitsch, Andrew P. King

**Affiliations:** King’s College London, The Rayne Institute, St Thomas’ Hospital, London, UK; European University of Cyprus, Nicosia, Cyprus

**Keywords:** Biomedical imaging, Positron emission tomography, Motion estimation, Magnetic resonance imaging

## Abstract

**Background:**

Respiratory motion in positron emission tomography (PET) is an unavoidable source of error in the measurement of tracer uptake, lesion position and lesion size. The introduction of PET-MR dual modality scanners opens a new avenue for addressing this issue. Motion models offer a way to estimate motion using a reduced number of parameters. This can be beneficial for estimating motion from PET, which can otherwise be difficult due to the high level of noise of the data.

**Method:**

We propose a novel technique that makes use of a respiratory motion model, formed from initial MR scan data. The motion model is used to constrain PET-PET registrations between a reference PET gate and the gates to be corrected. For evaluation, PET with added FDG-avid lesions was simulated from real, segmented, ultrashort echo time MR data obtained from four volunteers. Respiratory motion was included in the simulations using motion fields derived from real dynamic 3D MR volumes obtained from the same volunteers.

**Results:**

Performance was compared to an MR-derived motion model driven method (which requires constant use of the MR scanner) and to unconstrained PET-PET registration of the PET gates. Without motion correction, a median drop in uncorrected lesion ${\mathrm {SUV}}_{\mathrm {peak}}$ intensity to $78.4 \pm 18.6 \,\,\%$ and an increase in median head-foot lesion width, specified by a minimum bounding box, to $179 \pm 63.7\,\, \%$ was observed relative to the corresponding measures in motion-free simulations. The proposed method corrected these values to $86.9 \pm 13.6\,\, \%$ ($p<0.001$) and $100 \pm 29.12\,\, \%$ ($p<0.001$) respectively, with notably improved performance close to the diaphragm and in the liver. Median lesion displacement across all lesions was observed to be $6.6 \pm 5.4\,\mathrm {mm}$ without motion correction, which was reduced to $3.5 \pm 1.8\,\mathrm {mm}$ ($p<0.001$) with motion correction.

**Discussion:**

This paper presents a novel technique for respiratory motion correction of PET data in PET-MR imaging. After an initial 30 second MR scan, the proposed technique does not require use of the MR scanner for motion correction purposes, making it suitable for MR-intensive studies or sequential PET-MR. The accuracy of the proposed technique was similar to both comparative methods, but robustness was improved compared to the PET-PET technique, particularly in regions with higher noise such as the liver.

## Background

### Problems of motion in PET

Patient motion in PET has a detrimental effect on measured tracer uptake, lesion position and lesion size. This results in errors in detectability and quantification, leading to problems in disease staging, radiotherapy planning and other clinical/research PET applications [1].

Respiratory motion in particular is an important problem since the motion is involuntary and constant throughout the scan. The long duration of PET scans makes breath holding impractical: a typical PET scanner requires about 3 minutes of data acquisition per bed position (approximately $15$ to $20\,\mathrm {cm}$ axially). In [2], it was reported that maximal lung tumour displacement was observed to be as high as $22\,\mathrm {mm}$, with median displacement of $4.5\pm 5.0\,\mathrm {mm}$ across 22 subjects studied using radiography. This was verified by [3], who reported that tumours in the upper lung moved by up to $8.7\,\mathrm {mm}$, but that those in the bottom of the lung moved by up to $24.6\,\mathrm {mm}$. Other studies have shown that computed tomography (CT) visible lung lesions can move by up to $9\,\mathrm {mm}$, causing a variation in standardised uptake value (SUV, a measure of peak uptake value within a specified region) of up to 30 % [4], and that respiratory motion can lead to a mean overestimation of $1\,\mathrm {cm}$ diameter lesion volumes of 130 % [5].

Respiratory motion also leads to artefacts in PET-CT attenuation correction, which are created by mismatches between the emission and attenuation data. Artefacts typically appear as a band of artificially altered uptake above the diaphragm [6].

### Gating-based approaches

Over the last two decades, addressing this problem has been the focus of much research in the PET literature as well as in other applications. A common approach is amplitude gating, in which PET data are binned, or ‘gated’, based on the amplitude of a respiratory signal. This signal can be acquired using external hardware, such as pressure sensors, spirometry, temperature sensors, or video-tracking markers placed upon the patient’s thorax or abdomen [1]. However, acquiring a respiratory signal using these methods can be impractical and the correlation between the external signal and the internal motion is not always strong [7]. If the subject is in an MR scanner, an internal respiratory signal can be measured using a pencil-beam navigator, typically positioned on the diaphragm.

An alternative solution is the use of signals derived from the imaging data itself. Techniques have been proposed to derive respiratory signals from PET [8, 9], PET-CT [10, 11], or simultaneous PET-MR [12]. A comparative evaluation of some of these approaches has been presented by [13]. Signals derived using data-based methods are promising due to their direct relationship to the internal motion, but they depend heavily upon the quality of the imaging data.

After acquiring the respiratory signal, the simplest amplitude-gating approach is to reconstruct an image from a single gate and discard all data in the remaining gates. Whilst this approach reduces motion artefacts, each gate only has a proportion of the measured data, resulting in higher image noise than the full, ungated data [14].

### Motion correction

Motion correction techniques have been developed which make use of all available data rather than simply rejecting those which are motion-affected. This is likely to be advantageous for PET imaging, where the signal-to-noise ratio (SNR) is relatively poor.

Such techniques require an estimate of the motion undergone by the subject, which can then be used to transform all acquired data into a single image. In general a motion estimate describes the displacement of image features between a reference image and another image, and can take the form of a transformation matrix or vector field. In the latter case it is referred to as a motion field.

One approach is to estimate motion from the PET gates themselves. However, due to the high noise in individual gates, such estimates can lack accuracy and robustness [15, 16]. Techniques have been proposed for estimating motion fields from 4D CT [17, 18]. However, since this involves additional exposure to ionising radiation, CT can only be used to track motion for a limited time.

More recently, the introduction of simultaneous PET-MR scanners has opened up the possibility of using MR imaging to estimate subject motion during PET acquisition without the limitations imposed by radiation exposure. For example, in [19] MR images were registered to estimate motion transformations. These transformations were then matched to corresponding PET gates and used to transform them to a reference position. In [12], a respiratory signal was derived from MR and used to form gated MR images for correcting PET gates. In [20], gated MR images were formed from 2D slices using manifold alignment, and subsequently used for motion correction of simulated PET gates. These methods require sustained use of the MR scanner as a motion correction device. In [21], a technique was described that required less MR scanner time. A respiratory signal was derived from the PET data and used to gate the list–mode data into sinograms. The same signal was used to gate the MR data during a short, extra scan after the main PET acquisition. Motion was estimated from the MR gates and applied to the motion correct the PET gates.

Motion correction of PET data can be done in several ways. The two most straightforward are reconstruct-transform-average (RTA) [22] and motion-compensated image reconstruction (MCIR) [17, 18]. The former reconstructs and then motion corrects individual gates before combining them, whereas the latter incorporates motion correction into the PET reconstruction so that all available PET data are used in the reconstruction of a single, motion-corrected PET image. It has been reported that RTA introduces bias into the final image, whereas MCIR produces increased image noise [23]. This can, however, be reduced by appropriate regularisation techniques [24, 25]. A quantitative comparison of the RTA and MCIR techniques has recently been published using real PET-MR data [26].

In addition to motion correction of acquired PET data, motion estimates can be used to transform the attenuation map (e.g. CT), reducing artefacts due to mismatches between emission and attenuation data and removing the need for additional exposure from dynamic CT.

Respiratory motion is complex, nonlinear and subject to variation, both between breathing cycles (known as inter-cycle variation) and between the inhale/exhale phases of each cycle (known as intra-cycle variation, or hysteresis) [27]. Therefore these complexities make accurate and robust estimation of motion from gated images, particularly PET gates, challenging. It is important to note that, although many motion correction techniques have been proposed in the research literature, their clinical use is currently limited.

### Motion models

An alternative approach to estimating motion from gated images, which has gained increasing attention in the literature, is the use of a motion model. Motion models are mathematical functions which describe complex motion based on simpler input parameters, referred to as ‘surrogate’ signals. The technique we present in this paper is based on the use of a motion model, so we include here a brief introduction to the underlying theory and some notation. A known limitation of respiratory motion models is the implicit assumption that breathing during application does not significantly differ to that acquired during the calibration stage. The reader is referred to [27] for further details on motion models.

**Fig. 1:**
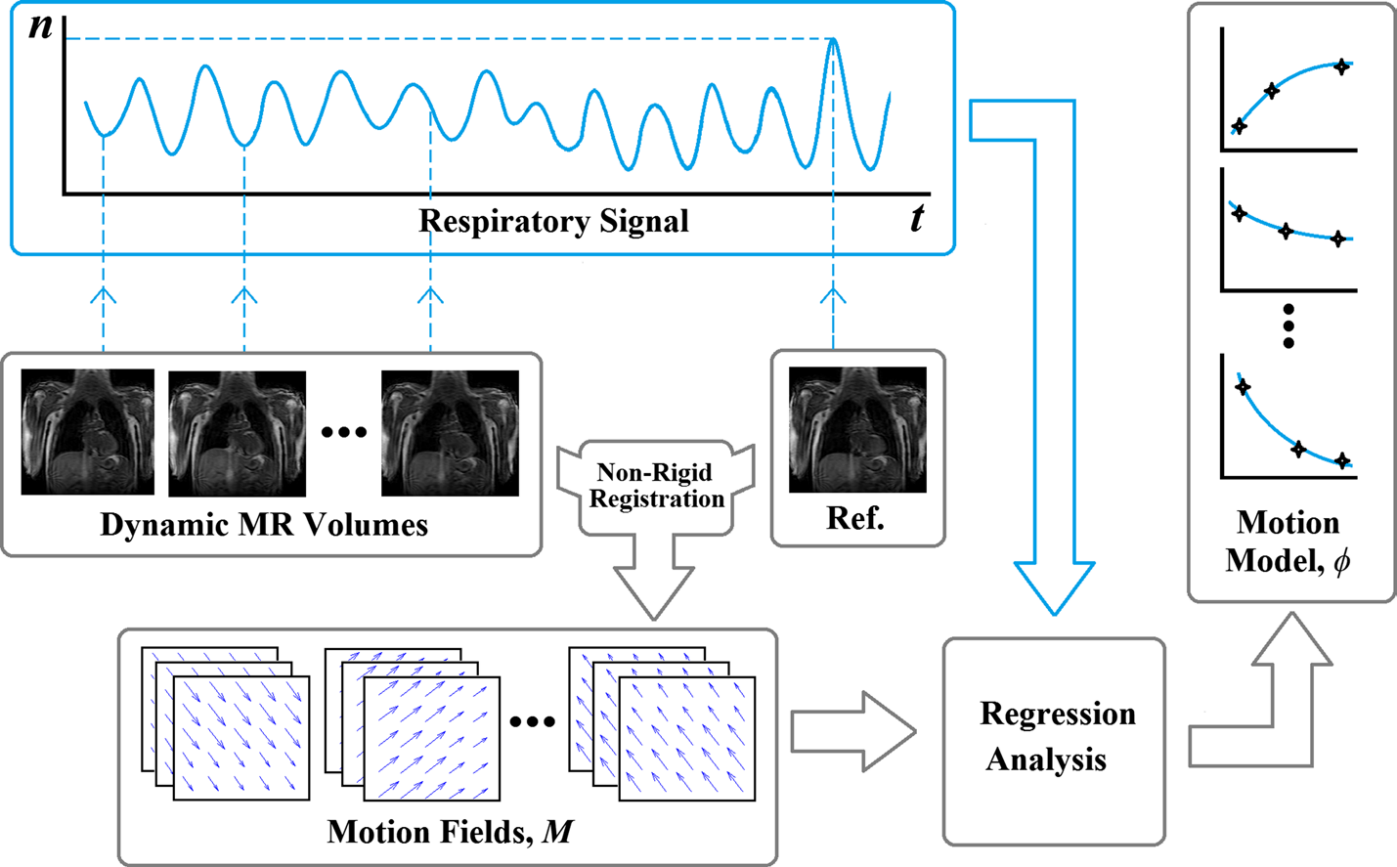
Forming a motion model from dynamic MR volumes. The MR volumes are registered to a reference volume to generate motion fields. These are used with the corresponding values of the respiratory signal (which act as the surrogate when forming the motion model) to produce a voxelwise model of the motion

To create a motion model, a number of samples of the motion are required. In the following discussion, we assume that these samples are voxelwise 3D vector fields known as motion fields. We denote these motion fields by $\mathbf {M}$, which parameterise the transformations between a reference position and other respiratory positions. Motion fields constitute the dependent variables of the motion model. A surrogate signal is typically acquired simultaneously with the motion sample acquisition scan and is used as the independent variable of the model. The motion model therefore captures the relationship between the surrogate signal and the motion fields. For an example of forming a motion model, refer to Fig. 1.

Traditionally, when applying a motion model to produce motion estimates, most authors have used a ‘direct’ correspondence model to link a measured surrogate, $\mathbf {s}$ (which may be a one or higher dimensional signal such as displacement of a point or surface), and the estimated motion fields $\mathbf {M}_\mathrm {DC}$:

1 $$\begin{aligned} \mathbf {M}_\mathrm {DC}=\mathbf \phi \left( \mathbf {s}\right) , \end{aligned}$$

where $\phi$ is the motion model.

An alternative approach is to employ an ‘indirect’ correspondence model [27]. In this case, the surrogate acquired during model application is not the same as that acquired during model formation (in fact often no surrogate is acquired during model formation). Rather, the surrogate used during model application is often a dataset of higher dimensionality (for example, an image). The model then makes use of a number of internal variables which are not measured directly. For example, in (2), an internal variable, $\mathbf {x}$, is optimised to maximise the similarity between the surrogate image transformed by a model-estimated motion field and a reference image,

2 $$\begin{aligned} \hat{\mathbf {x}}=\arg \max _{\mathbf {x}} \mathrm {Sim}\left\{ I_\mathrm {ref},\mathcal {T}\left[ \mathbf {s},\mathbf \phi \left( \mathbf {x}\right) \right] \right\} . \end{aligned}$$

where $\mathcal {T}$ is an operator which transforms the surrogate image, $\mathbf {s}$, using motion field estimate $\mathbf \phi \left( \mathbf {x}\right)$ and an internal variable, $\mathbf {x}$. This transformed image is compared to the reference image, $I_\mathrm {ref}$, using a similarity measure, $\mathrm {Sim}\left( \cdot \right)$.

In the case of a motion model describing respiratory motion, the internal variable, $\mathbf {x}$, could be correlated to the displacement of the diaphragm used in the formation of the model, but it is not required to measure $\mathbf {x}$ to apply the model. Rather, the motion fields for a given position are determined as a function of the surrogate image, $\mathbf {s}$.

The optimal values of the internal variables, $\hat{\mathbf {x}}$, are used as before to estimate the motion field

3 $$\begin{aligned} \hat{\mathbf {M}}_\mathrm {IC}=\mathbf \phi \left( \hat{\mathbf {x}}\right) . \end{aligned}$$

The indirect correspondence method for obtaining the transformations necessary for motion correction can also be viewed as a constrained image registration. The internal variable $\mathbf {x}$ is optimised to choose the transformation that best matches the surrogate image with the reference image.

Direct correspondence motion models have previously been proposed for motion correction in PET [28, 29]. However, such an approach would require the use of the MR scanner throughout PET scanning in order to acquire the necessary surrogate data (e.g. as a pencil-beam navigator). There has also been one example of an indirect correspondence motion model for PET motion correction, which used 2D MR images as surrogate data [30]. Although the results were promising, this approach would also require the use of the MR scanner throughout PET scanning.

To the authors’ knowledge, there has not yet been any use of an indirect correspondence motion model that has used the PET data themselves as the surrogate. If such an approach could be demonstrated to be effective, it would have a significant advantage in that the MR scanner would only need to be used to acquire a short calibration scan for motion model formation at the beginning of the simultaneous PET-MR scanning session. After this, the MR scanner would be free for clinical use. The approach would also have the added advantage of being compatible with sequential PET-MR. The application of an indirect correspondence model in this way results in one of the key novelties of our work: It offers a theoretical basis for enabling the use of both MR and PET data when estimating motion fields for PET motion correction. In this paper we describe and demonstrate such a technique, which uses an MR-derived motion model to constrain the range of possible PET-PET registrations, thus addressing the difficulties associated with estimating motion from low-SNR PET gates. Note that the PET data would still need to be gated. This can be achieved using the MR scanner, but there are alternative ways (some of which are deviceless), as discussed in “Gating-based approaches”. There have been very few joint MR+PET-based approaches in the literature (e.g. see [31, 32]).

In “Methods”, we describe our technique. “Experiments and results” describes evaluation using PET data simulated from real volunteer MR data, including real respiratory motion fields. The results are discussed in “Discussion”.

## Methods

In this section we present our proposed method. Specifically, we describe the formation of a respiratory motion model from dynamically-acquired MR volumes, and its application using an indirect correspondence model to constrain registrations between PET gates. We first provide details of the MR and PET imaging requirements in “Imaging requirements”. “Motion model formation” describes how MR images are used to form the motion model. “Motion model application” outlines the application of the MR derived motion model using an indirect correspondence model and PET gates as the surrogate data.

Note that throughout this section we use the term ‘surrogate’ to refer to the input to the motion model. To form the model, the surrogate is a simple respiratory signal derived from the MR images. When applying the model, the surrogate is the PET data, and the respiratory signal acts as an internal variable to be optimised.

### Imaging requirements

The MR imaging requirements for our proposed motion model are:

- a short dynamic 3D MR scan of the thorax during free breathing, resulting in a temporal sequence of near real-time 3D images (volumes) depicting the thoracic region at arbitrary respiratory motion states;
- a simultaneously-acquired respiratory signal. This will be used as the independent variable (i.e. surrogate) when forming the motion model, and it will be treated as the internal variable to be optimised during model application.

For information on specific acquisitions used in our experiments, refer to “MR data acquisition”.

The PET imaging requirement is that respiratory-binned PET gates are acquired using a gating technique. This could, for example, be based on an external signal [1] or make use of the PET data itself [11]. In this paper we evaluate our technique using simulated PET data and we provide details of our PET simulation procedure in “Simulating PET data from real MR images”.

### Motion model formation

The formation of the motion model is illustrated in Fig. 1. The first stage is to estimate respiratory motion from each dynamic MR volume. This is done by applying a nonrigid voxelwise registration [33] of the tth dynamic MR volume to the reference MR volume (corresponding to the most exhaled image, selected using the respiratory signal), which results in a motion field, $\mathbf {M}_t$. $\mathbf {M}_t$ is a matrix with a row for each voxel in the dynamic MR volume. The elements in each row represent the 3D displacement of that voxel. The displacement in each row is denoted by $\left( \Delta x_{ijk},\Delta y_{ijk},\Delta z_{ijk}\right)$, where *i*, *j*, *k* are the indices of the voxel in the volume.

Each dynamic MR volume has an associated value of the respiratory signal, $$n_t$$. These values are used as the independent variable in a regression analysis (i.e. the signal acts as the surrogate during model formation). The displacements $$\Delta x_{ijk}$$$$\Delta y_{ijk}$$ and $$\Delta z_{ijk}$$ ($$\forall ijk$$) are each modelled as separate 2nd-order polynomials of the respiratory signal value. The polynomial coefficients were determined by linear regression using the Vandermonde matrix method [34].

### Motion model application

To apply the model, the end-exhale PET gate is designated as the reference gate, $I_\mathrm {ref}$. The PET gate to be corrected, $I_g$, is transformed using motion fields produced by the motion model applied with trial values, $\left\{ x_i\right\}$, of a scalar internal variable, *x*. This scalar variable corresponds to the respiratory signal, $n_t$, that was used for model formation, and its trial values are evenly spaced between the minimum and maximum observed values of $n_t$. Since there is only one internal variable in this implementation, the optimisation is performed using 100 values in an exhaustive search. In more complex implementations, an automatic optimisation could be employed instead.

**Fig. 2:**
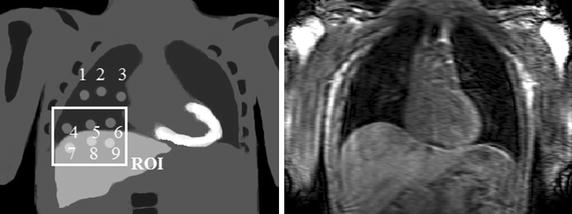
A coronal slice through a PET emission map showing the lesion positions (*left*) and an example of a dynamic MR volume (*right*). There are 9 lesions in total,* 3* in the top of the lung (*1*–*3*),* 3* above the diaphragm (*4*–*6*) and* 3* in the liver (*7*–*9*). These* numbers* will be used for referring to lesion positions. The VOI used for computing the similarity measure is also displayed. Note that the lesion position image is illustrative: only one lesion is present in each simulation

The resulting transformed image is compared to the reference PET gate. This comparison is performed in a volume of interest (VOI) placed over the lower right lung and the liver, as indicated in Fig. 2. In principle, the VOI could be any size or shape. This region was selected to maximise the contribution of high-contrast, high-motion regions to the similarity measure. This approach represents an exhaustive-search implementation of (2), using a scalar, *x*, as the internal variable $\mathbf {x}$, and the PET gate, $I_g$, as the surrogate image, $\mathbf {s}$. Equation (2) then becomes

4 $$\begin{aligned} \hat{x}=\arg \max _{x} \mathrm {Sim}\left\{ I_\mathrm {ref},\mathcal {T}\left[ I_g,\phi \left( x\right) \right] \right\} . \end{aligned}$$

**Fig. 3:**
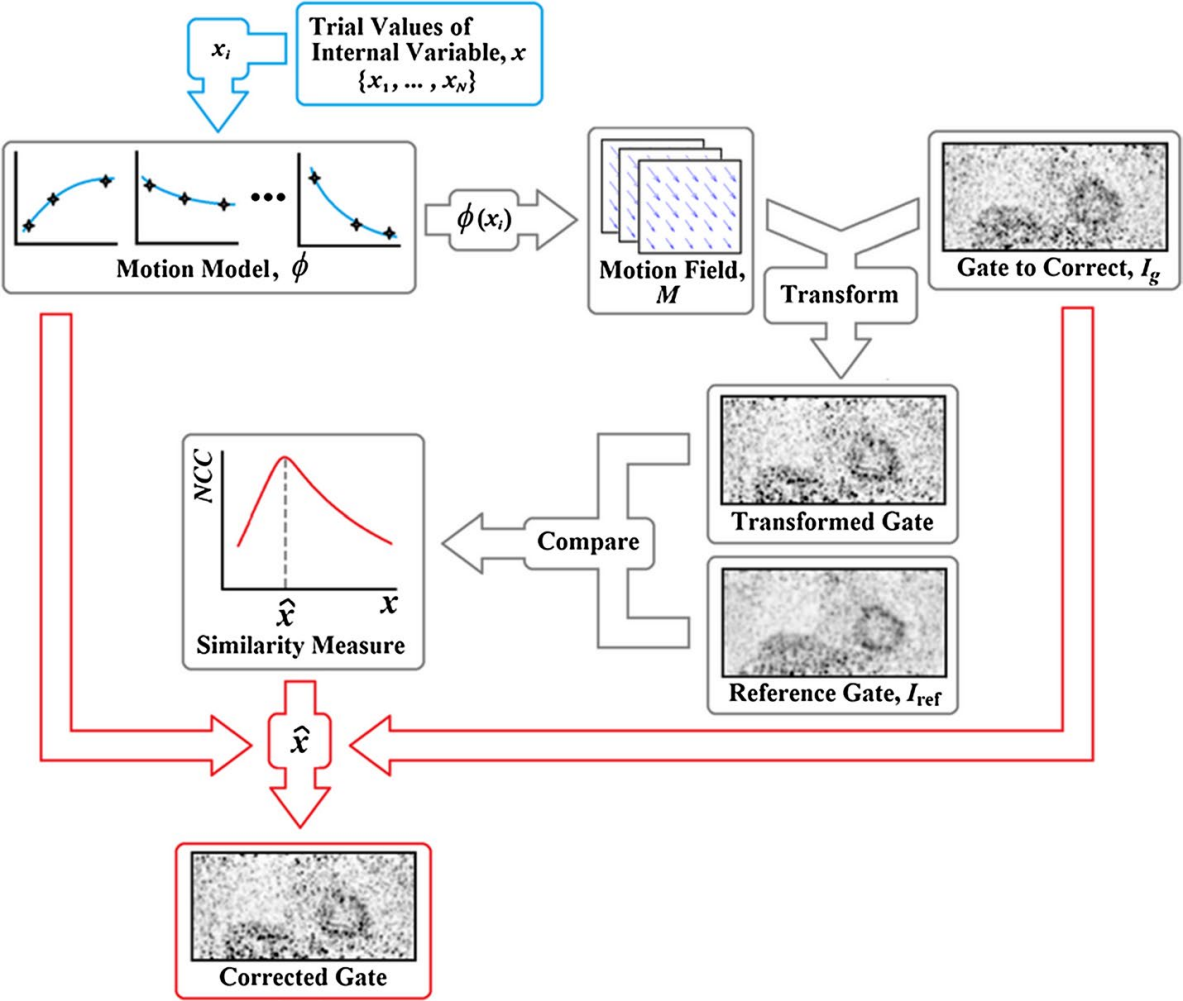
Schematic illustration of how the motion model is applied to motion correct a single gate of PET data, $I_g$. The input of a trial value for the internal variable, $x_i$, into the motion model, $\phi$, generates a trial motion field, $M$. The gate to be corrected is transformed using the motion field and quantitatively compared to the reference PET gate using a similarity measure. This process is repeated over a range of trial values for *x*. The value, $\hat{x}$, which produces the maximum similarity is then used with the motion model to motion correct $I_g$

Figure 3 illustrates how the motion model, $\phi$, is used in an indirect correspondence model approach to estimate the motion for each PET gate, $I_g$ (the same procedure is used for the other gates) and to use it for PET motion correction. The internal variable, *x*, of the model, $\phi$, takes the place of the respiratory signal *n* that was used in the model formation. The range of possible values of *x* is determined by the observed range of values of *n*, but the correct value for *x* is found by optimisation, so the respiratory signal does *not* need to be acquired to apply the model. This fact is an important feature of our technique and has a number of potential advantages that we discuss in “Discussion”.

All PET gates are blurred using a Gaussian smoothing filter prior to this procedure. We found in preliminary experiments that a Gaussian kernel with a standard deviation of $8\,\mathrm {mm}$ performed well. Note that this is only possible because the small-scale motion information is contained within the MR-derived motion model. Standard PET-PET methods would not have this freedom to reduce noise in the PET data without affecting motion estimation accuracy.

The similarity between the two images within the VOI is quantified by normalised cross correlation (NCC) [35]. NCC was chosen because amplitude-gated PET images have varying numbers of counts between gates, determined by the breathing pattern of the subject. Therefore, it is important to use a similarity measure that is less dependent on changes in image intensity.

The motion field that results in the maximum value of the similarity for each gate is selected as the optimal motion field, found using $\hat{\mathbf {M}}_g=\phi \left( \hat{x}_g\right)$. The final motion-corrected PET volume, $\tilde{I}_\mathrm {IC}$ is formed by applying the estimated motion fields to each of the original, unfiltered PET gates and averaging them according to RTA:

5 $$\begin{aligned} \tilde{I}_\mathrm {IC}=\sum ^G_{g=1}\mathcal {T}\left[ I_g,\phi \left( \hat{x}_g\right) \right] , \end{aligned}$$

where *G* is the number of PET gates acquired, including the reference gate.

## Experiments and results

To evaluate our proposed approach, we used simulated PET data created from real MR data. This allowed us to perform a quantitative evaluation of the performance of our technique using realistic motion fields. MR data was acquired from four healthy male volunteers (ages 22–33). Details of data acquisition are provided in “MR data acquisition”. The PET simulation procedure is described in “Simulating PET data from real MR images”. “Evaluation using simulated PET data” describes the evaluation of our technique using these data, and qualitative and quantitative results are presented in “Qualitative results” and “Quantitative results”.

### MR data acquisition

Two different MR sequences were acquired in the same scanning session for each volunteer. An ultrashort echo time (UTE) sequence was used to acquire images for forming emission and attenuation maps for PET simulation (see “Simulating PET data from real MR images”). A 3D dynamic sequence was used to acquire images for two purposes. Half of the data were used to calibrate the motion model (see “Motion model formation”). The other half were used to transform the emission and attenuation maps into real breathing positions for PET simulation. All data in these experiments were acquired using a Philips Achieva 3T MR scanner.

For the UTE sequence, two images were acquired in an interleaved fashion (each with different echo times) with respiratory gating, as described in [36]. The field of view was $400\times 400\times 400\,\mathrm {mm}$ at a resolution of $2\times 2\times 2\,\mathrm {mm}$, with $TR/TE_1=6.5/0.14\,\mathrm {ms}$ and $TE_2 = 4.6\,\mathrm {ms}$. A flip angle of 10° was used. Gating was achieved using a pencil-beam navigator positioned on the right hemidiaphragm. Scan duration was typically 10 to 30 min, depending on the subject’s breathing pattern and the efficiency of respiratory gating. The two resulting UTE images were subtracted to create a third image, which shows increased cortical bone contrast [36].

35 dynamic 3D MR volumes were acquired in quick succession for each subject during normal tidal breathing. These were used to estimate motion fields for model formation as described in “Motion model formation” and to transform the PET maps into real breathing positions. The sequence used to acquire the volumes was: T1-weighted FFE using SENSE protocol with SENSE factor 8, flip angle = $10^\circ$, field of view $500\times 450\times 245\,\mathrm {mm}$ with acquired image resolution $1.5\times 4.1\times 5\,\mathrm {mm}$ (FH, RL, AP) and reconstructed image resolution $1.5\times 1.5\times 5\,\mathrm {mm}$ and a time resolution of $0.7\,\mathrm {s}$ per image. For more information on this protocol, refer to [29]. An example of a single dynamic MR volume from this protocol is shown in Fig. 2.

The position of the right hemidiaphragm in the head-foot direction was estimated from each dynamic MR volume using cross-correlation of intensities within a manually-defined, cuboidal VOI [37]. This position value was used as the surrogate data, $n_t$, for motion model formation. It was subsequently optimised as the internal variable in model application, where the PET images themselves were considered to be the surrogate data of the motion model. Refer to “Motion model formation” and “Motion model application” respectively for more information on model formation and application. The respiratory signal was also used to select a reference end-exhale image for motion estimation.

Note that in our experiments the signal for model formation was image derived, but in practice it could easily be acquired as a pencil-beam navigator by the MR scanner.

### Simulating PET data from real MR images

The simulations were based on real anatomical and respiratory motion information derived from volunteer MR images, an approach which has been implemented in previous studies [36, 38].

The original UTE images and the difference UTE image (see “MR data acquisition”) were used to create two segmented maps for each volunteer. Segmentation was performed semi-automatically using ITK-SNAP [39]. These maps contained anatomical regions relevant to PET emission and attenuation respectively and formed the basis of the PET simulation, which is described briefly below. For a more in-depth explanation of this simulation procedure, refer to [38].

The simulation was intended to model a typical [^18^F]-fluorodeoxyglucose (FDG) scan, where tracer uptake is directly related to cellular glucose uptake. To study the effects of motion clearly, a spherical, FDG-avid lesion was inserted into the emission maps in addition to the other anatomical features. The lesion was positioned in nine different locations in the right thorax, with two different diameters: $10$ and $14\,\mathrm {mm}$. These are referred to as the ‘small’ and ‘large’ lesions respectively. The positions are shown in Fig. 2. Note that this figure is illustrative, and that each PET simulation only contained one lesion in one of these positions. Separate emission and attenuation maps were produced for each lesion size/position combination, and separate PET simulations performed for each.

Each emission/attenuation map was transformed to different motion positions using the transformations derived by registering dynamic MR volumes. As described in “MR data acquisition”, 35 dynamic MR volumes were acquired for each volunteer, corresponding to approximately 6 breathing cycles. For each volunteer, 17 volumes were used for simulating PET gates. The remaining unused 18 volumes were used to create the motion model for motion correction. The volumes were assigned to either PET simulation or model formation according to order of acquisition, but with the constraint that a similar distribution of respiratory states should be present within each group. This ensured that separate data were used for producing the PET test data and for forming the motion model in our experiments. The most-exhaled volume of the 35 (based on the value of the respiratory signal, see “MR data acquisition”) was chosen as the reference volume. All other volumes were nonrigidly registered [33] to this reference volume to generate motion fields between each volume and the reference volume. The right hemidiaphragm in volunteers 1, 2, 3, and 4 was observed to displace by up to $25.2$, $20.7$, $13.3$, and $38.7\,\mathrm {mm}$ respectively.

Each PET simulation was binned into respiratory gates, with the gates corresponding to 6 equally-spaced intervals within the observed range of respiratory signal values. The 17 motion fields were separated into these 6 bins based on the values of their respiratory signals, $n_t$. In these experiments, we used $n_t$ to perform the binning for convenience, but in reality any other suitable gating technique could be used (e.g. [1, 11]). The emission/attenuation maps for each PET gate were generated by transforming the segmented maps using each motion field within the corresponding bin, and then averaging all transformed maps. This averaging approximates the blurring artefacts introduced by the motion that would occur within each PET gate during continuous data acquisition. Note that motion modelling was *not* used during this simulation procedure.

The averaged, transformed maps were used to simulate realistic PET images with the Software for Tomographic Image Reconstruction (‘STIR’, [40]). Following the method described in [38], the images were modelled as being acquired on a Phillips Gemini TF, with a reconstructed voxel size of $2\times 2\times 2\,\mathrm {mm}^3$. Prior to simulation, emission maps were smoothed with a $4\,\mathrm {mm}$, isotropic Gaussian filter to approximate resolution effects. A total of 50 million counts were simulated for each scan (typical for a 5 min FDG scan), with each gate having a fraction of these proportional to the period each volunteer was observed to spend in each respiratory gate. These fractions were computed by counting the number of volumes (out of the 35) within each gate based on their respiratory signal value. Finally, each gated sinogram was individually reconstructed using the ordered-subsets expectation maximisation algorithm (23 subsets, 10 iterations).

A total of 72 motion-included simulations were created (9 lesion positions, 2 diameters and 4 volunteers). Some examples of the simulated PET gates can be seen in Fig. 3. Note the different noise characteristics in each image due to different count totals in the gated sinograms. The ‘ideal’ motionless (ML) image was also created for each simulation, with the same physical effects and processes. For this, the simulation procedure was identical to the simulations involving motion, except each ‘motionless’ gate was the reference PET gate used in the motion-included simulations, repeated 6 times (i.e. once for each PET gate), each simulated with the same aforementioned noise levels proportional to breathing amplitude. This was done to maintain any effects of reconstruction bias resulting from using the RTA technique. These motionless simulations allow characterisation of best achievable performance of all methods presented in this study.

### Evaluation using simulated PET data

To characterise the performance of our proposed indirect correspondence model based method (IC) in correcting for the effects of motion on the simulated PET data, we compared it to three alternative approaches to combining multiple PET gates:

- *Uncorrected (UC)*: All 6 motion-affected PET reconstructed gates were averaged without including any motion correction.
- *Direct correspondence model (DC)*: The same motion model as we have described in “Motion model formation” was employed to motion correct the simulated PET gates, but a direct correspondence model based technique was used for model application. Specifically, the MR image-derived respiratory signal described in “MR data acquisition” was used directly as the surrogate input to the model to estimate the motion field for each gate (i.e., in (1), the respiratory signal, *n*, was used as the surrogate, $\mathbf {s}$). This is the technique described in [29] and would require continuous use of the MR scanner to acquire the pencil-beam navigator.
- *Unconstrained PET-PET Registration (PT)*: Nonrigid registration [33] was used to register each PET gate to the reference PET gate. The estimated motion fields were used to motion correct each gate and the transformed gates were subsequently averaged.

We used visual inspection plus three methods of quantifying the performance of our technique, which represented peak lesion uptake, width, and position relative to the equivalent measure in the motionless (ML) image.

#### Peak lesion uptake value

The first quantitative measure was the percentage correction of lesion peak uptake value relative to the corresponding value in the motionless simulations. Peak lesion uptake values of the lesions were calculated as $\mathrm {SUV}_{\mathrm {peak}}$. $\mathrm {SUV}_{\mathrm {peak}}$ was found by using a small VOI (in this case, a central voxel and its 6 nearest neighbours) and taking the mean voxel value of the small volume. This average was then attributed to the central voxel. The voxel with the highest mean defined in this way within a larger VOI was designated as the $\mathrm {SUV}_{\mathrm {peak}}$ of the lesion. For this work, the larger VOI was a user-defined volume of approximately $20\times 20\times 20\,\mathrm {mm}$ centred on the location of each lesion in the motionless image. Manual definition was required in some cases to avoid noise features within the image. Intensity recovery was quantified as a percentage of $\mathrm {SUV}_\mathrm {peak}$ of each lesion, with 100 % defined as the respective value in the motionless simulation.

#### Lesion width

Respiratory motion can cause significant changes in apparent lesion volume and shape. This is seen predominantly in the head-foot (HF) direction since this is the primary direction of displacement for respiratory motion. However, the use of profiles through the lesion does not adequately characterise the shape of the activity distribution. This makes full–width at half–maximum (FWHM) derived from line profiles a poor quantifier of PET motion correction.

Instead, a minimal bounding box was defined within the VOI used to find $\mathrm {SUV}_{\mathrm {peak}}$ of each lesion. This box minimally fit the whole surface defined by the lesion’s FWHM in the 3 directions of the image planes. This was automated using Matlab, then checked manually. The automation algorithm created a 3D binary image around each given lesion, in which voxels were assigned a value of 1 if their intensity value was equal to or greater than half the value of the peak intensity. The algorithm then identified the smallest box required to enclose the binary image. The widths of the box were then used to quantify lesion width in each of the three image directions: head-foot, anterior-posterior, and left-right. Lesion width recovery was defined as a percentage of the size of the minimum bounding box in the motionless simulations, in each direction.

#### Lesion position

Finally, the error in lesion position was computed as the third measure. This was done by calculating the magnitude of the 3D displacement of the lesion $\mathrm {SUV}_{\mathrm {peak}}$ relative to the motionless case.

Note that different Poisson noise realisations were used for each simulation (i.e. the seed for the random number generator was selected randomly between 1 and 1000 for each PET gate), so even if perfect motion correction transformations were applied there would be differences in noise character between any given lesion and its motionless equivalent.

### Qualitative results

#### Visual inspection

**Fig. 4:**
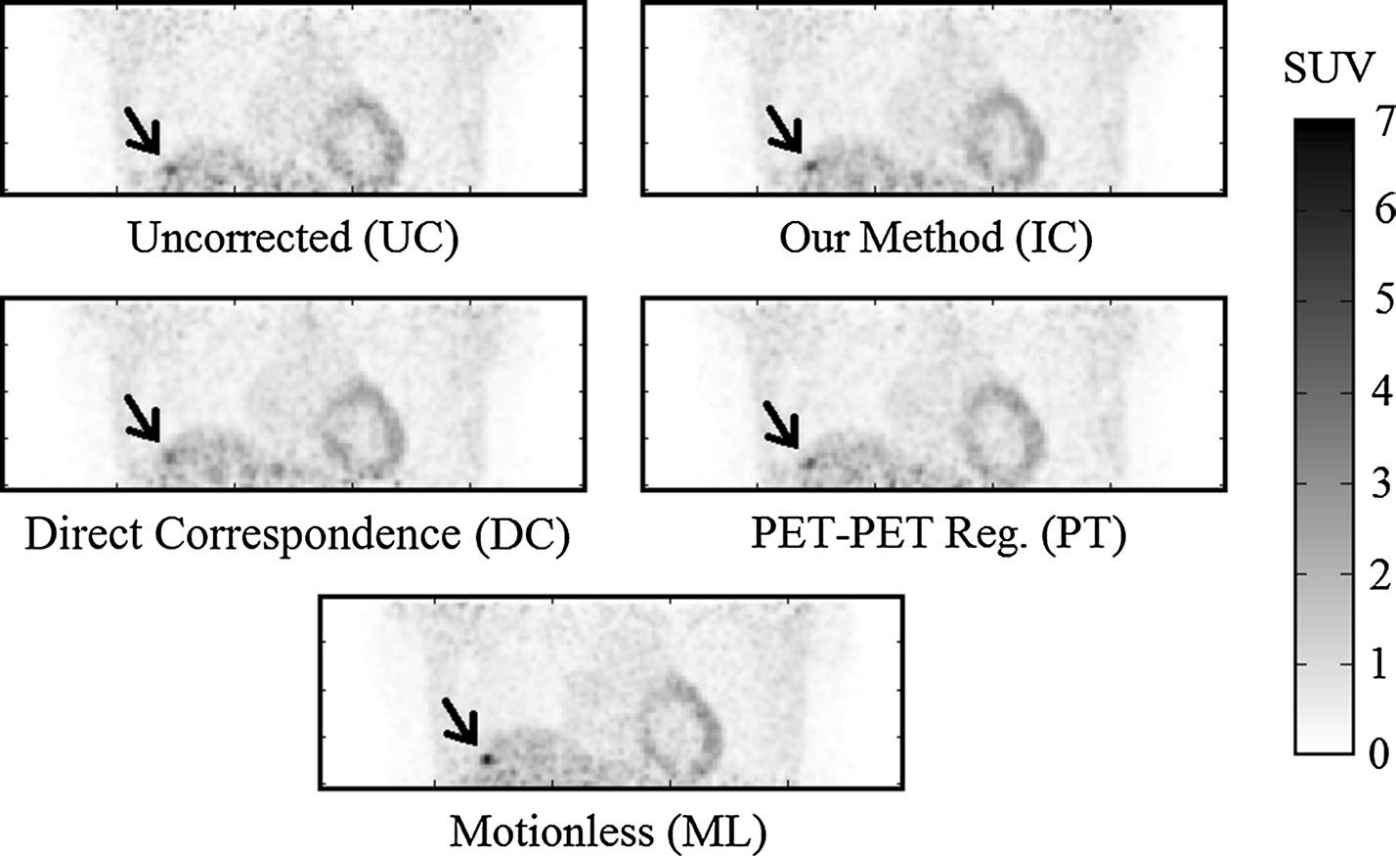
Coronal views of a small lesion, position 4 for volunteer 2, displayed with a $4\,\mathrm {mm}$ Gaussian filter. The intensity scale is shown to the *right*. The lesion (indicated with an *arrow*) is relatively indistinct in the UC case, but clear in the IC case. In this example, the PET-PET registration (PT) has performed moderately well, whereas the direct correspondence method (DC) has performed poorest of the 3 correction methods. Also notice the blurred appearance of the liver-lung boundary in the UC image

**Fig. 5:**
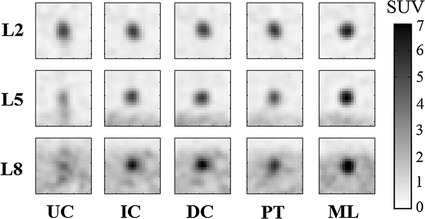
The effects of motion correction on large lesions in positions 2, 5, and 8 in coronal views of volunteer 4. Refer to Fig. 3 for lesion positions. Effects are shown for the uncorrected PET (UC), the indirect correspondence model proposed in this paper (IC), the direct correspondence method from [29] (DC), unconstrained PET-PET registration (PT) and the gold-standard set by motionless PET (ML). Note that L2 does not move much. This agrees with the observation reported by [3] that lesions in the upper lung move by around $2\,\mathrm {mm}$: below PET image resolution. However, L5 and L8 have lost contrast due to respiratory motion, which is recovered with varying success by correction methods IC, DC and PT. Profiles of these lesions are displayed in Fig. 6

**Fig. 6:**
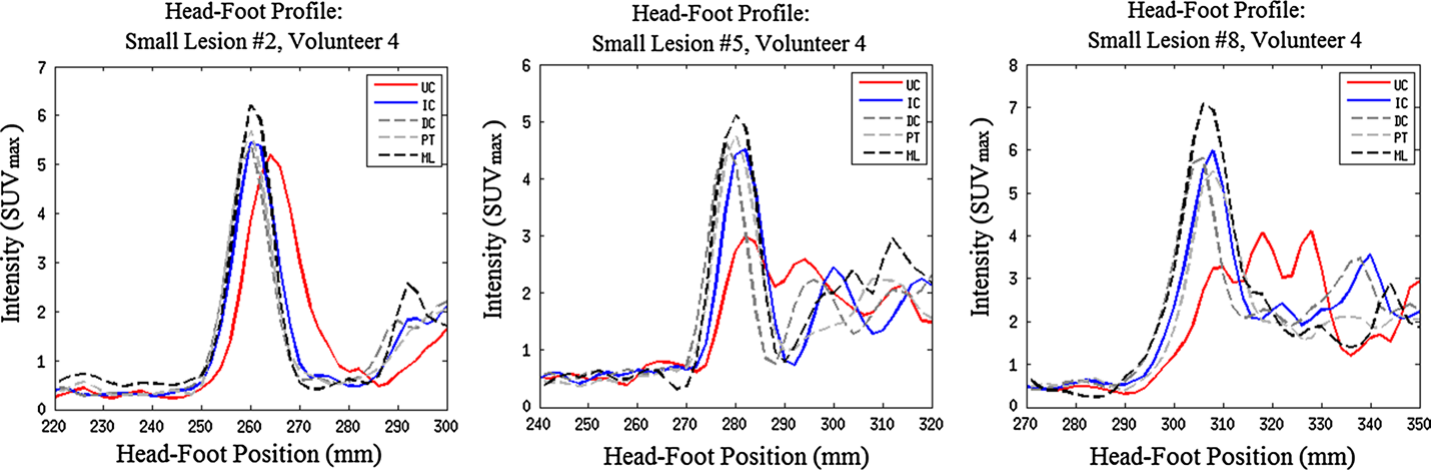
Head-foot profiles through large lesions in positions 2, 5, and 8 respectively for volunteer 4 Coronal slices of these lesions are displayed in Fig. 5. Notice the similarity of all profiles for the lung lesion (2, *left*), but the significant spreading of the UC lesion profile on the diaphragm (5, *centre*) and in the liver (8, *right*). In all cases, the IC, DC, and PT correction methods recover a significant proportion of the ML peak uptake value

Some results of applying these three approaches, together with the ‘ideal’ motionless (ML) simulation are shown in Figs. 4, 5 and 6. Visually, our proposed approach and both comparative techniques improve the visibility of the lesions. In Fig. 4, which displays a small lesion in position 4, the uncorrected PET image has an indistinct patch of increased activity within the right inferior lobe of the lung. It is questionable whether this would be identified as a lesion by a clinician inspecting this scan. Upon motion correction, this patch becomes a distinct, higher contrast lesion above the diaphragm. Compared to the motionless case, the motion correction techniques qualitatively recover lesion size and position, but not full contrast.

### Quantitative results

**Fig. 7:**
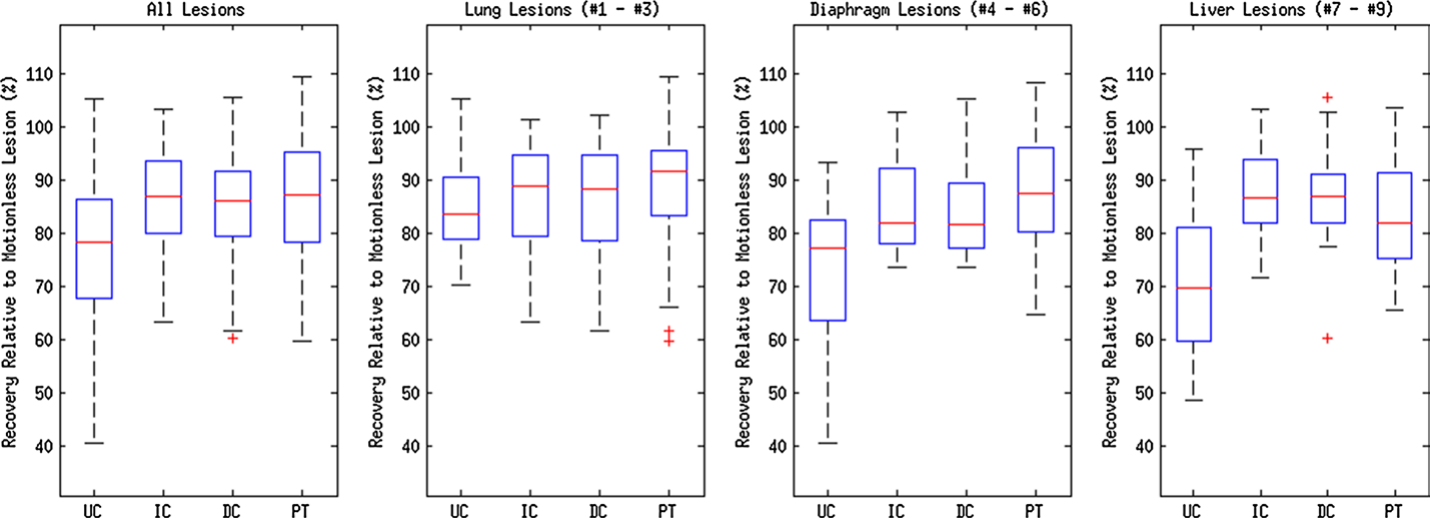
Peak uptake value as a percentage of equivalent motionless lesion $\mathrm {SUV}_\mathrm {peak}$ as a *box plot*. Labels correspond to: (UC) Uncorrected PET, (IC) Our proposed indirect correspondence technique, (DC) The direct correspondence technique, and (PT) unconstrained PET-PET registration

**Fig. 8:**
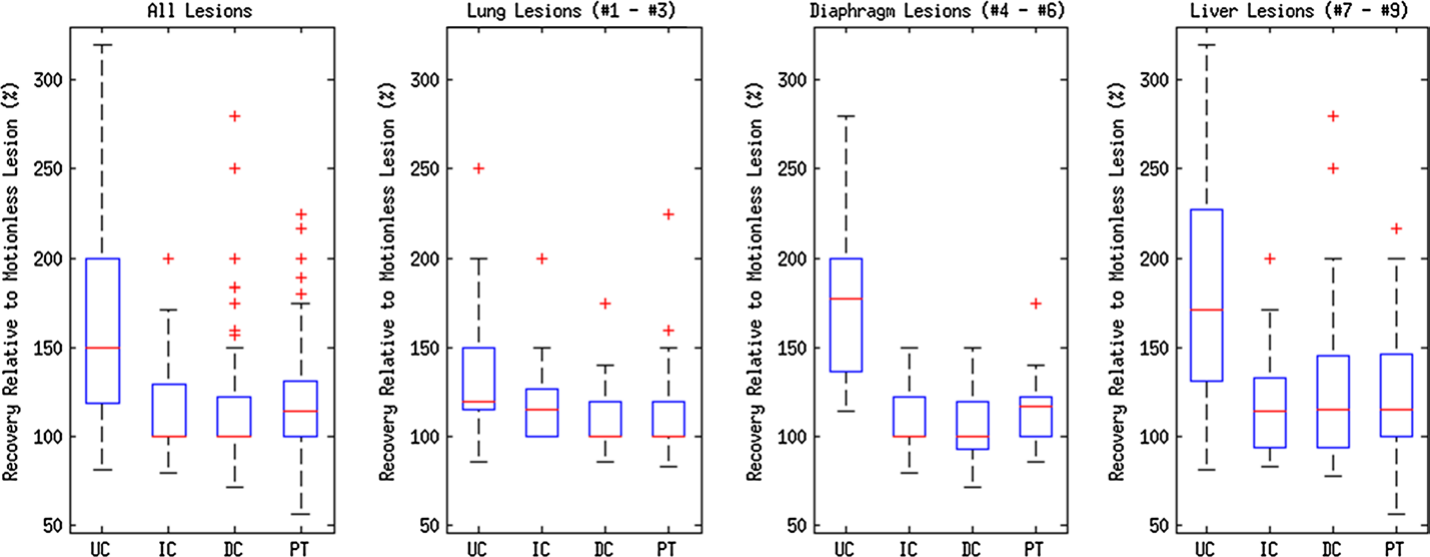
Width of the minimum bounding box of each lesion in the head-foot direction, as a *box plot*. Note that widths are quoted as a percentage of the corresponding motionless lesion value. Labels correspond to: (UC) Uncorrected PET, (IC) Our proposed indirect correspondence technique, (DC) The direct correspondence technique, and (PT) Direct PET-PET registration. Note that for IC and DC in the All Lesions graph and DC in the Lung Lesions graph, the median corresponds to the lower quartile

**Fig. 9:**
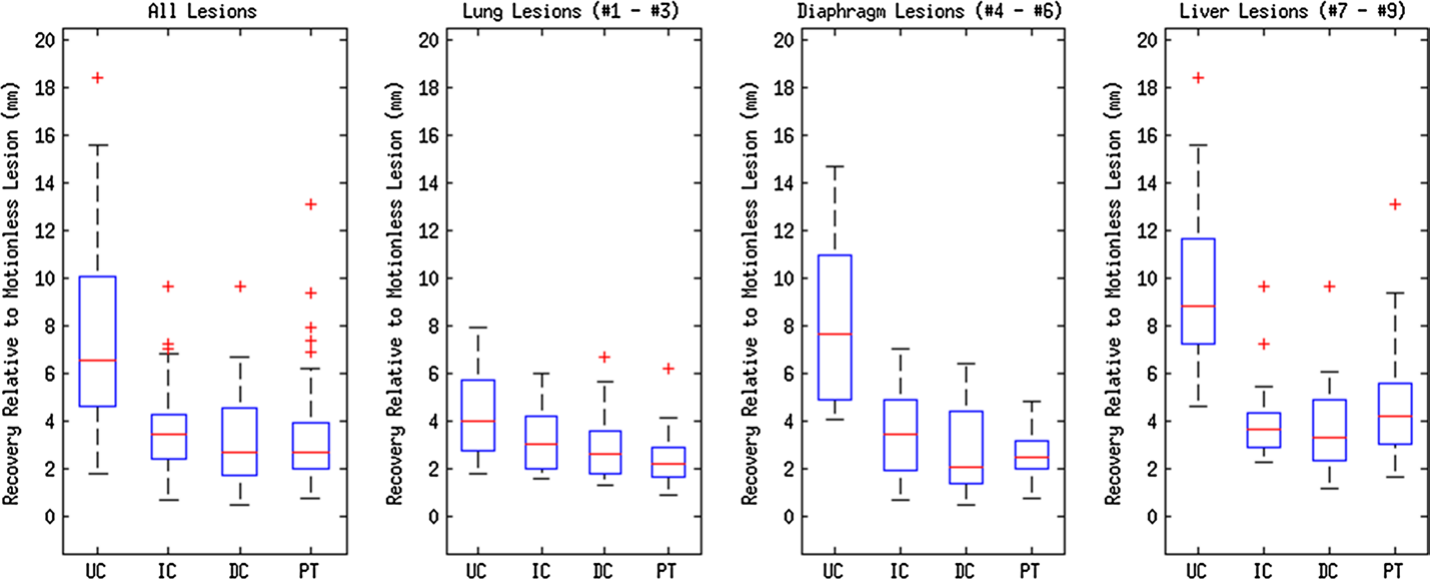
Displacement of each lesion centroid in millimetres as a* box plot*. Labels correspond to: UC, uncorrected PET; IC our proposed indirect correspondence technique; DC the direct correspondence technique; PT direct PET-PET registration

Quantitative results for our experiments are shown in Figs. 7, 8 and 9. Note that all results are quoted in median and interquartile range since skew was observed in the data distributions. A 2–tailed Wilcoxon signed rank test was applied to test the statistical significance of the results in all experiments. Tests were performed to compare all motion correction techniques to the uncorrected case. A value of $p\le 0.01$ (i.e. 99 % confidence) was taken as a statistically-significant result.

#### Peak lesion uptake value

Recovery of peak uptake value was found using $\mathrm {SUV}_{\mathrm {peak}}$. These values are percentages with the motionless lesion at 100 %. Values will thus generally be lower than 100 %, although sometimes they may be slightly higher due to image noise.

The distribution of changes in $\mathrm {SUV}_{\mathrm {peak}}$ compared to the motionless case are presented as box and whisker plots in Fig. 7. In the ‘All Lesions’ graph of Fig. 7, the overall changes in $\mathrm {SUV}_\mathrm {peak}$ of each method are displayed: uncorrected (UC) PET, PET motion corrected with the indirect correspondence technique proposed in this paper (IC), direct correspondence motion model (DC), and direct application of a nonlinear registration algorithm to each PET gate (PT). Subsequent graphs in the figure correspond to each region of the thorax with simulated lesions: the lung (i.e. lesions 1 to 3), diaphragm (lesions 4 to 6) and liver (lesions 7 to 9).

Uncorrected, all lesions were observed to only recover a median of $78.4\pm 18.6\,\, \%$ of the motionless lesion $\mathrm {SUV}_{\mathrm {peak}}$. The indirect correspondence motion modelling method proposed in this paper recovered a median of $86.9 \pm 13.6\,\, \%$ ($p=1.9\times 10^{-9}$), whereas the direct correspondence application of the same motion model yielded $86.3\pm 12.1\,\,\%$ ($p=9.9\times 10^{-6}$) of the motionless $\mathrm {SUV}_{\mathrm {peak}}$ peaks. Nonlinear registration of the PET gates recovered $87.2\pm 16.9\,\,\%$ ($p=3.8\times 10^{-7}$) of the peak intensities on average.

**Fig. 10:**
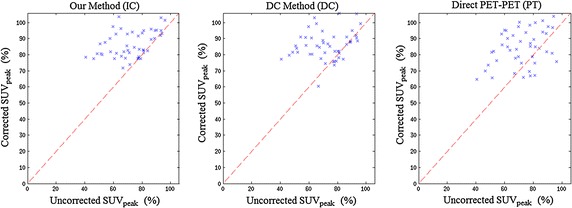
Illustrating method robustness with scatter plots of $\mathrm {SUV}_\mathrm {peak}$ recovery. These scatter plots show the improved robustness of the two MR-derived motion model based techniques (DC and IC) compared to the direct registration PET-PET registration technique (PT). Each plot compares distributions of $\mathrm {SUV}_\mathrm {peak}$ for each lesion in positions 4–9 for all volunteers, both before correction and after correction by the IC, DC and PT methods. The* dashed line* represents $y=x$. Any point on this *line* was unaffected by the correction attempt. Points above the *line* have had their SUVpeak increased (with 100 % corresponding to the motionless peak uptake), whereas points below the *line* show a reduction in SUVpeak. Note in particular that whilst none in the IC case are significantly below the *line*, 8 lesions dropped in SUVpeak with the PT method (16.7 % of the points shown)

The lowest observed uncorrected lesion intensity was 40.6 % of noiseless activity. The IC, DC, and PT methods recovered this to 78.4, 64.8, and 77.3 % respectively. The scatter plots in Fig. 10 show the behaviour of individual lesion measurements before and after correction. The dashed line $y=x$ defines the point at which no improvement is achieved. Points below this line have worsened under the correction method, whilst those above have improved. These plots are discussed further in “Robustness analysis”.

#### Lesion width

The changes in the head-foot width of the minimum bounding box for each lesion FWHM are shown in Fig. 8. Once again, the respective motionless lesion profile was used to define 100 %. Contrasting to the results for correction in peak uptake value, the values in Fig. 8 tend to be greater than 100 %.

Overall, the uncorrected PET images exhibited a median increase in width to $150 \pm 82\,\, \%$ of that of the motionless lesions. The IC motion correction reduced this to $100 \pm 29\,\, \%$ of the motionless lesion width ($p=4.4\times 10^{-15}$). Similarly, the DC motion correction technique reduced this to $100 \pm 23\,\, \%$ ($p=3.4\times 10^{-11}$), whereas the direct PET-PET registration reduced the minimal bounding box width to $114\pm 30\,\,\%$ ($p=3.9\times 10^{-15}$).

#### Lesion position

The displacement magnitudes of lesions from their motionless positions are presented in Fig. 9. Note that the original position of a lesion is at $0\,\mathrm {mm}$. Uncorrected PET lesions showed a median offset of $6.6\pm 5.4\,\mathrm {mm}$. The IC method reduced this median to $3.5\pm 1.8\,\mathrm {mm}$ ($p=2.4\times 10^{-17}$). The DC method reduced the median displacement to $2.7\pm 2.8\,\mathrm {mm}$ ($p=2.0\times 10^{-15}$). The PT method reduced the median displacement to $2.7\pm 1.9 \,\mathrm {mm}$ ($p=3.6\times 10^{-18}$).

### Robustness analysis

**Fig. 11:**
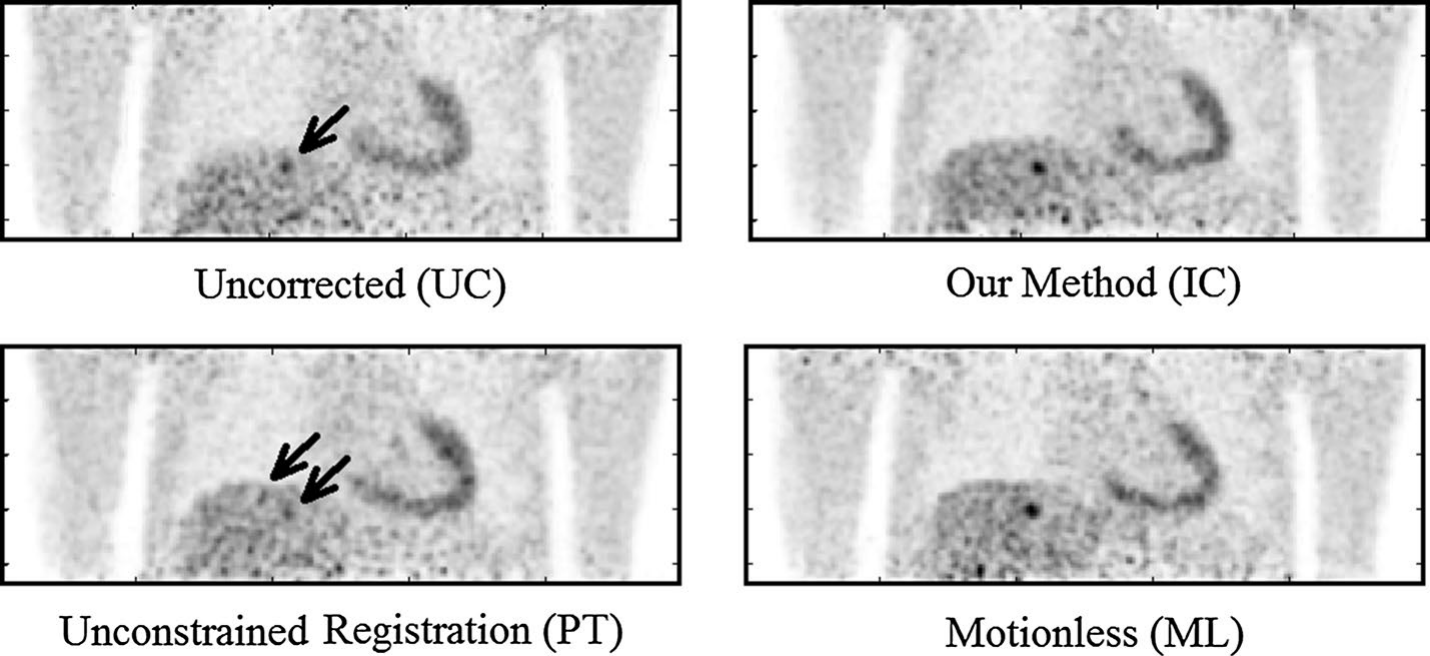
A case in which image quality deteriorates after unconstrained PET-PET registration based motion correction. Here, the PT method has failed to recover a small lesion in position 9 of volunteer 1. As can be seen, a small, indistinct lesion is indicated by an arrow in the UC image. This is clearly recovered using our method (IC) to a visual quality comparable to the motionless case. However, unconstrained registration (PT) has actively made the lesion harder to see, with two possible candidates indicated by* arrows*. These are comparable to the levels of noise seen elsewhere in the liver, and could easily be missed. All images are shown on the same intensity scale

The IC technique matched the results achieved by the DC technique, therefore it should be able to achieve correction in any situation for which the DC technique has already been tested, such as those in [29]. Similarly, the IC technique matched unconstrained PET-PET registration, but was more robust in certain cases:

- The range of errors on each PT all-lesion average are larger than those in the IC case, with (occassionally many) outliers on the box plots in Figs. 7, 8, and 9.
- The PT method underperformed for most liver lesions, and in some cases lesions were not recovered, such as in Fig. 11. There are no cases where a lesion was notably worse due to the IC technique, as can be seen by the scatter plots in Fig. 10. Note that this latter figure only shows lesions in positions 4–9 for clarity; the lung lesions (positions 1–3) are mostly unaffected by the attempts at motion correction due to their small displacement as a result of respiratory motion, and so fluctuate around $y=x$.
- Registration for the IC and DC cases are constrained to realistic biomechanical positions, as defined by the MR. The same cannot be said for the PT method, especially when a greater number of respiratory gates are used, due to lower SNR.

## Discussion

We have demonstrated a novel technique for motion correction of PET gates that would not require sustained use of the MR scanner during a simultaneous PET-MR scanning session. Performance was comparable with an MR-derived motion model technique that would require such use of the MR scanner. Experiments indicated that overall our technique could recover median lesion peak uptake value up to $86.9 \pm 13.6\,\,\%$ (from $78.4 \pm 18.6\,\,\%$ for uncorrected PET), and median lesion size down to $100 \pm 15.7\,\,\%$ (from $179 \pm 63.7\,\,\%$ in uncorrected PET) of the affected head-foot width. Lesion displacements were improved from $6.6\pm 5.4\,\mathrm {mm}$ prior to correction to $3.5\pm 1.8\,\mathrm {mm}$. All of these improvements were statistically significant—below the $p=0.01$ threshold.

Whilst the average lesion intensity only improved by an additional 8 %, this could be due to the depth of breathing of the volunteers. The maximal observed displacements of the right hemidiaphragm in volunteers 1, 2, 3, and 4 were $25.2$, $20.7$, $13.3$, and $38.7\,\mathrm {mm}$ respectively. Since the lesion sizes were $10$ and $14\,\mathrm {mm}$, these displacements span 1 to 3 times the lesion size, which could cause different behaviour under motion.

### Advantages

The indirect correspondence motion model based technique has some advantages over other motion correction techniques. The main advantage is that the motion correction only requires the MR scanner for a short, initial motion model calibration scan to provide a method for robust PET-PET registration. After this scan, the motion model can be formed and applied without any further requirement of the MR scanner, maximising its availability for other clinical or research purposes. This independence from MR also makes this method of respiratory motion correction suitable for sequential PET-MR. It may also have the potential to correct for some spatiotemporal misalignments between the PET and MR coordinate systems. In addition, errors in surrogate signal acquisition for direct correspondence model based methods are avoided. This final point could explain why the IC method occasionally outperformed the DC method in our experiments, resulting in a larger spread of values for lesions corrected by the DC method. Note that our technique does still require a respiratory signal to be measured during MR scanning (for model formation) and also during PET scanning (for gating purposes). However, the two signals do not need to be the same. The requirement to acquire the same or a similar signal, which is a feature of many alternative techniques (such as [28, 29]), introduces several potential difficulties. First, if the signal is measured using the MR scanner, it restricts the use of the technique to simultaneous PET-MR, and furthermore it limits the use of the scanner for clinical purposes during PET scanning. Second, if the signal is measured using an external device such as an optical or magnetic tracker the measurement device must be MR-compatible and any line-of-sight issues must be resolved. Use of an external signal also increases the cost of the solution and complicates clinical work flows. The fact that our technique eliminates this requirement is therefore an important feature, and could result in greatly simplified clinical work flows. In addition, using the PET data itself as the surrogate enables our technique to use information about PET-visible lesions in the motion correction process, potentially improving motion correction accuracy in areas of clinical interest. However, the biomechanical constraints of motion model based techniques are preserved, leading to more robust results than the direct PET-PET registration technique.

### Relation to other work

The technique we have described represents a novel approach to PET motion correction, but has some similarities with related approaches from the literature. Several papers have recently proposed the use of MR to obtain motion estimates to correct PET [12, 19]. These works registered MR gates to directly obtain motion estimates, which were then used to motion correct gated PET images prior to averaging them [12, 19]. A similar approach was taken by Manber et al [21], in which the respiratory signal used for gating was derived from the PET data. This allowed the MR scanner to be free for clinical use, apart from a short calibration scan (similar to our technique). However, in Manber et al’s work, the PET data were not directly used in the motion estimation procedure. In our work, the PET data are directly employed in the motion estimate, but this is constrained using an MR-derived motion model.

4D registration approaches (e.g. [41]) involve a similar ‘constrained registration’ approach, in that sets of transformations that are not smooth between gates are effectively excluded from the motion estimation process. However, our approach uses constraints estimated from another, more reliable, modality (i.e. MR). We do not currently use between-gate smoothness as a constraint, but this would be an interesting extension of our technique.

Other related works include [31], who used a probabilistic model based on a mixture of Gaussians to make use of MR data to jointly estimate activity and motion parameters in a single PET reconstruction. More recently, [32] used a combined MR and PET similarity measure when registering gated MR and PET images.

### Limitations and future improvements

The motion fields used in our PET simulations were derived from real MR scans. The motion should therefore be very realistic. However, deriving motion from low resolution dynamic 3D MR scans has known weaknesses: in particular, contrast inside the lungs can be poor. Thus motion fields inside the lungs are, effectively, interpolated from those at the high-contrast lung boundaries. This fact makes our results for lung lesions (i.e. in positions 1–3) less reliable than those close to the diaphragm (positions 4–9). Also note that the UC errors in the lungs are smaller than in the other regions. This is likely due to the unreliable motion fields as well as the smaller magnitude of motion in this region.

Our method uses a region of interest to compute the similarity measure from the PET gates. This volume is extended across the lower lung and much of the liver. In our experiments, a lesion in positions 4 through 9 could therefore provide additional information to the IC constrained registration, which could aid the registration process. In contrast, lesions 1 to 3 are outside of this region of interest. We used a smaller region to maximise the proportion of high-contrast structure within the VOI. This made it easier to estimate the optimal value of the internal variable, $\hat{x}$. Theoretically, the IC registration would find the same motion fields for each of these 3 lesion positions (i.e. 1 to 3) for any given volunteer, since the anatomical information available is identical. However, the noise characteristics of each simulation differ, causing fluctuations in the registration result. Due to this, the IC method performs slightly differently for each lung lesion.

In our experiments, perfect attenuation correction was assumed. Attenuation was included in the simulations, but anatomically accurate attenuation maps were assumed to be known for each respiratory position. This allowed us to focus our evaluation on the effects of our algorithm on motion correction alone. In future work, we plan to incorporate our approach into an MCIR-based motion correction algorithm. This will necessitate formulating the optimisation of an iterative reconstruction to update the internal respiratory signal values for each gate. It would then be relatively straightforward to incorporate attenuation correction into the reconstruction procedure in such a way that this assumption is no longer required.

Moving towards an MCIR approach has a number of other benefits. Firstly, MCIR is desirable due to its improved ability to provide quantitatively-accurate results. Since iterative PET reconstruction is an optimisation process, it naturally fits with the optimisation part of the indirect correspondence motion model. Moreover, the low SNR of gated sinograms becomes less of a problem as it will not introduce bias into the reconstruction (a limitation imposed by the non-negativity constraint of iterative reconstruction algorithms).

The motion model we employed in this work was a relatively simple ‘average-cycle’ model, which would not be able to capture any intra-cycle motion variation and only limited inter-cycle variation. Use of this simple model allowed us to demonstrate a proof-of-principle for our proposed motion correction approach, but more sophisticated types of motion model could result in improvements in performance. For example, multiple internal variables could be employed, such as signals derived from other anatomical positions like the chest/abdomen or even statistical dimensionality reduction approaches such as principle component analysis [11].

A further limitation of our work is the fact that the MR data used for model formation and PET simulation were acquired in the same scan. It would be more realistic to acquire the data in separate scans to simulate the long scan time of PET imaging. We also plan to evaluate our technique on real PET data in the future.

Our technique used an indirect correspondence model approach using PET gates as the surrogate. We compared this with a direct correspondence model approach using an MR-based respiratory signal as the surrogate, but an alternative approach would have been to use a respiratory signal derived from PET data as the surrogate (e.g. [10, 11]). However, such an approach would introduce uncertainty as to how to relate the different (but similar) surrogate signals used to form the model (i.e. MR-based signal) and apply it (PET-based signal). Our approach has no such problem since we effectively optimise the value of the signal used to form the model based on the richer information contained in the PET gates used as surrogate images.

## Conclusion

The proposed method represents a proof-of-principle of what the authors believe to be a new class of PET motion correction techniques. More complex implementations, using some of the improvements outlined in the previous section, are possible. In addition, this technique is one of the first attempts to use both PET and MR data to estimate motion fields for PET motion correction, which the authors believe is a potentially fruitful area for future research.

In summary, we believe that the technique we have described represents an important addition to the literature on PET-MR motion correction, in that we have demonstrated that good, reliable PET motion correction performance can be achieved without continuous or repeated use of the MR scanner. This potentially makes incorporating motion correction into clinical protocols much more feasible.

## References

[1] Nehmeh SA, Erdi YE (2008). Respiratory motion in positron emission tomography/computed tomography: a review. Semin Nucl Med.

[2] Stevens CW, Munden RF, Forster KM, Kelly JF, Liao Z, Starkschall G, Tucker S, Komaki R (2001). Respiratory-driven lung tumor motion is independent of tumor size, tumor location, and pulmonary function. Int J Radiat Oncol Biol Phys.

[3] Seppenwoolde Y, Shirato H, Kitamura K, Shimizu S, van Herk M, Lebesque JV, Miyasaka K (2002). Precise and real-time measurement of 3D tumor motion in lung due to breathing and heartbeat, measured during radiotherapy. Int J Radiat Oncol Biol Phys.

[4] Erdi YE, Nehmeh SA, Pan T, Pevsner A, Rosenzweig KE, Mageras G, Yorke ED, Schoder H, Hsiao W, Squire OD (2004). The CT motion quantitation of lung lesions and its impact on PET-measured SUVs. J Nucl Med.

[5] Liu C, Pierce LA, Alessio AM, Kinahan PE (2009). The impact of respiratory motion on tumor quantification and delineation in static PET/CT imaging. Phys Med Biol.

[6] Beyer T, Antoch G, Blodgett T, Freudenberg LF, Akhurst T, Mueller S (2003). Dual-modality PET/CT imaging: the effect of respiratory motion on combined image quality in clinical oncology. Eur J Nucl Med Mol Imaging.

[7] Zeng R, Fessler JA, Balter JM, Balter PA (2008). Iterative sorting for four-dimensional CT images based on internal anatomy motion. Med Phys.

[8] Visvikis D, Barret O, Fryer T, Turzo A, Lamare F, Cheze le Rest C, Bizais Y. A posteriori respiratory motion gating of dynamic PET images. In: Nuclear Science Symposium Conference Record, 2003 IEEE, 2003; vol. 5, pp 3276–3280. doi:10.1109/NSSMIC.2003.1352596

[9] Bundschuh RA, Martínez-Moeller A, Essler M, Martínez MJ, Nekolla SG, Ziegler SI, Schwaiger M (2007). Postacquisition detection of tumor motion in the lung and upper abdomen using list-mode PET data: a feasibility study. J Nucl Med.

[10] Schleyer PJ, O’Doherty MJ, Barrington SF, Marsden PK (2009). Retrospective data-driven respiratory gating for PET/CT. Phys Med Biol.

[11] Thielemans K, Rathore S, Engbrant F, Razifar P. Device-less gating for PET/CT using PCA. In: Nuclear Science Symposium and Medical Imaging Conference (NSS/MIC), 2011 IEEE, 2011, pp 3904–3910. doi:10.1109/NSSMIC.2011.6153742

[12] Grimm R, Fürst S, Souvatzoglou M, Forman C, Hutter J, Dregely I, Ziegler SI, Kiefer B, Hornegger J, Block KT, Nekolla SG (2015). Self-gated MRI motion modeling for respiratory motion compensation in integrated PET/MRI. Med Image Anal.

[13] Thielemans K, Schleyer P, Marsden PK, Manjeshwar RM, Wollenweber SD, Ganin A. Comparison of different methods for data-driven respiratory gating of PET data. In: Nuclear Science Symposium and Medical Imaging Conference (NSS/MIC), 2013 IEEE, 2013; pp 1–4. doi:10.1109/NSSMIC.2013.6829055

[14] Boucher L, Rodrigue S, Lecomte R, Bénard F (2004). Respiratory gating for 3-dimensional PET of the thorax: feasibility and initial results. J Nucl Med.

[15] Dawood M, Lang N, Jiang X, Schafers KP (2006). Lung motion correction on respiratory gated 3-D PET/CT images. IEEE Trans Med Imag.

[16] Bai W, Brady M (2009). Regularized B-spline deformable registration for respiratory motion correction in PET images. Phys Med Biol.

[17] Qiao F, Pan T, Clark JW, Mawlawi OR (2006). A motion-incorporated reconstruction method for gated PET studies. Phys Med Biol.

[18] Li T, Thorndyke B, Schreibmann E, Yang Y, Xing L (2006). Model-based image reconstruction for four-dimensional PET. Med Phys.

[19] Würslin C, Schmidt H, Martirosian P, Brendle C, Boss A, Schwenzer N, Stegger L (2013). Respiratory motion correction in oncologic PET using T1-weighted MR imaging on a simultaneous whole-body PET/MR system. J Nucl Med.

[20] Baumgartner CF, Kolbitsch C, Balfour DR, Marsden PK, McClelland JR, Rueckert D, King AP (2014). High-resolution dynamic MR imaging of the thorax for respiratory motion correction of PET using groupwise manifold alignment. Med Image Anal.

[21] Manber R, Thielemans K, Hutton BF, Barnes A, Ourselin S, Arridge SCO, Wan S, Atkinson D (2015). Practical PET respiratory motion correction in clinical PET/MR. J Nucl Med.

[22] Picard Y, Thompson CJ. Motion correction of PET images using multiple acquisition frames. IEEE Trans Med Imag 1997;16(2) 10.1109/42.563659 9101323

[23] Polycarpou I, Tsoumpas C, Marsden PK (2012). Analysis and comparison of two methods for motion correction in PET imaging. Med Phys.

[24] Chun SY, Fessler JA (2013). Noise properties of motion-compensated tomographic image reconstruction methods. IEEE Trans Med Imag.

[25] Tsoumpas C, Polycarpou I, Thielemans K, Buerger C, King AP, Schaeffter T, Marsden PK (2013). The effect of regularization in motion compensated PET image reconstruction: a realistic numerical 4D simulation study. Phys Med Biol.

[26] Fayad H, Schmidt H, Würslin C, Visvikis D (2015). Reconstruction incorporated respiratory motion correction in clinical simultaneous PET/MR imaging for oncology applications. J Nucl Med.

[27] McClelland JR, Hawkes DJ, Schaeffter T, King AP (2013). Respiratory motion models: a review. Med Image Anal.

[28] Reyes M, Malandain G, Koulibaly PM, González-Ballester MA, Darcourt J (2007). Model-based respiratory motion compensation for emission tomography image reconstruction. Phys Med Biol.

[29] King AP, Tsoumpas C, Buerger C, Schulz V, Marsden P, Schaeffter T. Real-time respiratory motion correction for simultaneous PET-MR using an MR-derived motion model. In: Nuclear Science Symposium and Medical Imaging Conference (NSS/MIC), 2011 IEEE, 2011; pp 3589–3594. doi:10.1109/NSSMIC.2011.6153674

[30] King AP, Buerger C, Tsoumpas C, Marsden PK, Schaeffter T (2012). Thoracic respiratory motion estimation from MRI using a statistical model and a 2-D image navigator. Med Image Anal.

[31] Pedemonte S, Bousse A, Hutton BF, Arridge S, Ourselin S. 4-D generative model for PET/MRI reconstruction. In: Fichtinger G, Martel A, Peters T (eds.) Med Image Comput Comput Assist Interv. Lecture Notes in Computer Science, vol. 6891, Springer 2011; pp 581–588. 10.1007/978-3-642-23623-5_73 22003665

[32] Fieseler M, Gigengack F, Jiang X, Schafers K (2014). Motion correction of whole-body PET data with a joint PET-MRI registration functional. BioMed Eng Online.

[33] Buerger C, Schaeffter T, King AP (2011). Hierarchical adaptive local affine registration for fast and robust respiratory motion estimation. Med Image Anal.

[34] Horn RA, Johnson CR. Matrix analyis, Reprint edn., p. 29. Cambridge University Press, Cambridge 1990.

[35] Hill DLG, Batchelor PG, Holden M, Hawkes DJ (2001). Medical image registration. Phys Med Biol.

[36] Buerger C, Tsoumpas C, Aitken A, King AP, Schleyer P, Schulz V, Marsden PK, Schaeffter T. Investigation of MR-based attenuation correction and motion compensation for hybrid PET/MR. IEEE Trans Nucl Sci 2012;59(2).

[37] Savill F, Schaeffter T, King AP. Assessment of input signal positioning for cardiac respiratory motion models during different breathing patterns. In: Biomedical Imaging: From Nano to Macro, 2011 IEEE International Symposium On, 2011; pp 1698–1701. doi:10.1109/ISBI.2011.5872731

[38] Tsoumpas C, Buerger C, King A, Mollet P, Keereman V, Vandenberghe S, Schulz V, Schleyer P, Schaeffter T, Marsden PK (2011). Fast generation of 4D PET-MR data from real dynamic MR acquisitions. Phys Med Biol.

[39] Yushkevich PA, Piven J, Cody Hazlett H, Gimpel Smith R, Ho S, Gee JC, Gerig G (2006). User-guided 3D active contour segmentation of anatomical structures: significantly improved efficiency and reliability. Neuroimage.

[40] Thielemans K, Tsoumpas C, Mustafovic S, Beisel T, Aguiar P, Dikaios N, Jacobson MW (2012). STIR: software for tomographic image reconstruction release 2. Phys Med Biol.

[41] Klein GJ, Reutter RW, Huesman RH (2001). Four-dimensional affine registration models for respiratory-gated PET. IEEE Trans Nucl Sci.

